# Editorial: Perspectives in pharmacological therapy targeting cellular metabolic pathways in respiratory diseases

**DOI:** 10.3389/fphar.2023.1324586

**Published:** 2023-11-27

**Authors:** Na Wang, Jie Liu, Peiran Yang, Dustin Fraidenburg, Haiyang Tang

**Affiliations:** ^1^ Department of Pulmonary and Critical Care Medicine, Shanghai East Hospital, Tongji University School of Medicine, Shanghai, China; ^2^ Key Laboratory of Arrhythmias of the Ministry of Education of China, Department of Cardiovascular Surgery, Research Center for Translational Medicine, Shanghai Heart Failure Research Center, Shanghai East Hospital, Tongji University School of Medicine, Shanghai, China; ^3^ State Key Laboratory of Respiratory Health and Multimorbidity, Department of Physiology, Institute of Basic Medical Sciences, Chinese Academy of Medical Sciences and School of Basic Medicine, Peking Union Medical College, Beijing, China; ^4^ Department of Medicine, University of Illinois at Chicago, Chicago, IL, United States; ^5^ State Key Laboratory of Respiratory Disease, National Clinical Research Center for Respiratory Disease, Guangzhou Institute of Respiratory Health, The First Affiliated Hospital of Guangzhou Medical University, Guangzhou, Guangdong, China

**Keywords:** respiratory disease, metabolism, metabolic pathway, idiopathic pulmonary fibrosis, acute lung injury, chronic obstructive pulmonary disease, lung infection

Interventions targeting metabolic pathways show promise for innovative therapeutic approaches and disease management. We established a Research Topic, entitled “Perspectives in Pharmacological Therapy Targeting Cellular Metabolic Pathways in Respiratory Diseases” to delve into the most recent advancements in respiratory disease treatment. This Research Topic features contributions from 82 authors with 10 manuscripts and one abstract, along with over 10,000 views and 3,000 article downloads, spanning from basic research to clinical applications. The articles include original research, reviews, bioinformatic analysis, clinical cases, and studies, providing a comprehensive insight on managing various pulmonary diseases, including idiopathic pulmonary fibrosis (IPF), acute lung injury (ALI), lung infections, chronic obstructive pulmonary disease (COPD), pulmonary malignancies, and pulmonary edema.

IPF is a debilitating lung disease characterized by progressive scarring and fibrosis of the lung tissue. Excessive collagen deposition and tissue remodeling are significant hallmarks of IPF (Raghu et al., 2018), both associated with heat shock protein 27 (HSP27) which is a major trigger for epithelial-to-mesenchymal transition (EMT) (Kim et al., 2019). Yoo et al. presented a novel HSP27 inhibitor, NA49, and tested it in radiation and bleomycin-induced pulmonary fibrosis models. NA49 treatment effectively attenuated fibrosis development by hindering NF-κB signaling and EMT. Notably, NA49 caused less DNA damage in human lung epithelial cells compared to previous HSP27 inhibitors. With enhanced pharmacokinetics, treatment with NA49 stands out as a promising approach in pre-clinical pulmonary fibrosis models. This study presents impressive progress in our pharmacological modulation of HSP27, evolving from antisense oligonucleotides to a new generation of small molecule inhibitors (Wettstein et al., 2013). Regarding the metabolism-immunology interplay in IPF development, Yang et al. performed bioinformatic analysis and differentiated two subtypes with distinct metabolic phenotypes. IPF patients in subtype C1 exhibited less active nucleotide, fatty acid, and amino acid metabolism, correlating with a poorer prognosis than C2. C1 also exhibited increased CD8^+^ T cell, macrophage, and neutrophil infiltration, alongside upregulated cytokines, chemokines, and TGF-β levels. A diagnostic model comprising nine genes was established. Compound analysis suggested adenosine receptor-targeting meds for C1 and glucose-lactic acid-targeting meds for C2, paving the way for tailored IPF treatments.

Mitochondria, a pivotal cellular organelle, governs numerous metabolic pathways including carbon, lipid and amino acids metabolism. Proper mitochondrial function is imperative for generating essential ATP via oxidative phosphorylation (Sharma et al., 2021), while dysfunctional mitochondria may lead to compromised ATP production and surges in reactive oxygen species (ROS) generation, resulting in overactivated mitophagy (Ornatowski et al., 2020). Tian et al. reported that caffeine effectively attenuates high-altitude pulmonary edema (HAPE) and improves lung function in HAPE rats by enhancing mitochondrial oxidative phosphorylation (OXPHOS) and mitochondrial quality control in AT1 cells. Furthermore, caffeine boosted electron transport chain (ETC.) activity, increasing ATP production and mitochondrial bioenergetics. It also promoted glycolytic enzymes, offering an alternative energy source under hypoxia. Caffeine further enhanced mitophagy via the PINK1/Parkin pathway, facilitating damaged mitochondria disposal. Together, these findings underscore caffeine’s potential in alleviating HAPE through the optimization of mitochondrial function and quality control.

Endoplasmic reticulum (ER) stress interacts with metabolic processes through the unfolded protein response (UPR), which monitors protein folding within the ER. The UPR modulates various metabolic pathways, including lipid metabolism, energy homeostasis, inflammation, and cell differentiation (Hetz, 2012). Chang et al. presented the effects of compound B6 on cigarette smoke-induced inflammation and injury in bronchial epithelial cells. The study revealed that B6 mitigated ER stress and inflammation by reducing the expression of BIP, ATF4, and CHOP, suggesting B6 as a potential therapeutic option for COPD-related inflammation and injury. Lou et al. reported Urolithin A (UA) as a prospective treatment for acute lung injury (ALI) induced by lipopolysaccharide in mice. UA exhibited efficacy by enhancing the Keap1-Nrf2/HO-1 pathway, reducing oxidative stress, and ameliorating lung damage, suggesting UA as a potent anti-inflammatory and antioxidant agent in ALI treatment.

Pulmonary infections pose a substantial global health concern, necessitating timely pathogen identification and antibiotic treatment (Magill et al., 2014). Conventional diagnostic approaches often fall short, particularly in the face of multiple infections. In contrast, metagenomic next-generation sequencing (mNGS) has emerged as a commendable leap in clinical microbiology. Han et al. evaluated mNGS for diagnosing pulmonary infections involved 101 participants. The results showed that both Illumina and BGI mNGS platforms outperformed conventional methods in sensitivity, with negligible differences between each platform. This implies that mNGS can enhance pulmonary infection diagnosis by providing earlier pathogen identification, honing treatment precision, and enriching epidemiological insights. The rising incidence and mortality of fungal infections, including pulmonary mycosis, underscore the urgency for the demand of precise diagnosis and efficient treatment (Maitre et al., 2021). Bronchoscopic instillation has been hailed as a novel therapeutic approach for pulmonary mycosis, offering precise local targeting of lesions. However, there has been a lack corroborated clinical data, specific indicators, or parameters gauging the efficacy of this strategy for patients (Lang et al., 2020). Yang et al. conducted a study, involving 80 patients, examined bronchoscopic instillation of amphotericin B. The results showed 72.5% of patients experienced imaging improvements, 77.5% manifested imaging or localized containment of mycosis improvements, and 95% exhibited imaging improvements and containment or fell within the immunotherapy timeframe. This approach demonstrated a commendable success rate coupled with minimal adverse events, spotlighting its potential, particularly for systemic antifungal non-responders or those unable to tolerate it.

Non-small-cell lung cancer (NSCLC) is one of the most common cancers worldwide and causes a vast number of cancer-related deaths (Siegel et al., 2022). SMARCA4, a tumor suppressor gene, is reportedly mutated in approximately 10% of NSCLC patients and associated with poor prognosis (Hodges et al., 2018). While immune checkpoint inhibitors have been used to treat NSCLC, immune-related adverse events can be challenging to manage and may limit the use of these therapeutic agents (Atchley et al., 2021). In their report, Deng et al. shared their experiences regarding a patient with SMARCA4-mutant NSCLC, who developed immune-related pneumonitis during anti-PD-1 treatment and chemotherapy. Switching therapies to corticosteroids and nintedanib, an anti-fibrotic multi-tyrosine kinase inhibitor, led to the patient’s recovery and allowed chemotherapy to resume. This study illustrates an interesting strategy of using an anti-fibrotic agent to manage immune-related adverse events and encourages further research on the applications of nintedanib with immune checkpoint inhibitors in NSCLC.

In summary, this Research Topic provides an exhaustive insight into the current state of research related to the diagnosis and management of respiratory diseases. It highlights pioneering therapeutic targets and the emergence of novel pharmaceutical agents in the field. This Research Topic of studies underscores not only the depth of existing knowledge but also illuminates pathways toward new therapeutic strategies. Our aspiration is to catalyze further researches, particularly in translational applications, fostering the evolution of more refined and efficacious strategies to improve patient outcomes in clinical practice.
